# Isolated left ventricular apical hypoplasia: a case report

**DOI:** 10.1093/ehjcr/ytaf650

**Published:** 2025-12-23

**Authors:** Ujjwala Tulluri, Tilak Suvarna, Ganesh Barhate

**Affiliations:** Asian Heart Institute and Research Centre- Department of Cardiology, Department of Radiology, G/N, G Block BKC, Bandra Kurla Complex, Bandra East, Mumbai, Maharashtra, India 400051; Asian Heart Institute and Research Centre- Department of Cardiology, Department of Radiology, G/N, G Block BKC, Bandra Kurla Complex, Bandra East, Mumbai, Maharashtra, India 400051; Asian Heart Institute and Research Centre- Department of Cardiology, Department of Radiology, G/N, G Block BKC, Bandra Kurla Complex, Bandra East, Mumbai, Maharashtra, India 400051

**Keywords:** Isolated left ventricular apical hypoplasia, Cardiomyopathy, Cardiac imaging, Adult congenital heart disease, Heart failure, Case report

## Abstract

**Background:**

Isolated left ventricular apical hypoplasia is a rare and lesser-known form of cardiomyopathy characterized by specific findings on cardiac imaging. It is thought to be congenital in origin, with clinical features that can range from being asymptomatic to presenting with heart failure and arrhythmias.

**Case summary:**

A 43-year-old woman presented with chest pain and had previously undergone evaluation at another facility, including a 2D echocardiogram and computed tomography (CT) coronary angiography. These tests revealed a dilated left atrium and ventricle and reduced left ventricular function alongside a Type I left anterior descending artery. A cardiac magnetic resonance image (MRI) and review of the CT scan confirmed a diagnosis of isolated left ventricular apical hypoplasia. Guideline-directed medical therapy was initiated due to the initial presentation of left ventricular dysfunction.

**Conclusion:**

Left ventricular apical hypoplasia is characterized by (i) a truncated, spherical, and impaired left ventricle (LV) with bulging of the interventricular septum towards the right ventricle (RV), (ii) fatty material in the apical region of the LV, (iii) abnormalities in the papillary muscles and trabecular structures, and (iv) elongation of the RV wrapping around the underdeveloped LV. Limited data is available on this condition, with only a few reported cases. Currently, no definitive guidelines exist, and management is tailored to the patient’s specific presentation, including treating heart failure and any arrhythmias that may arise. While the aetiology of this condition remains poorly understood, it is crucial to recognize it to prevent misdiagnosis and to encourage further research into its management.

Learning points

- Isolated left ventricular apical hypoplasia (ILVAH) is a rare cardiomyopathy defined by a distinctive left ventricular morphology and variable clinical presentation.
- Cardiac MRI is the diagnostic modality of choice, characteristically demonstrating a truncated, spherical left ventricle with apical fat replacement and right ventricular encasement.
- No standardized management guidelines exist; treatment is individualized, focusing on heart failure optimization and arrhythmia control.

## Introduction

Isolated left ventricular apical hypoplasia (ILVAH) is a rare and distinct cardiomyopathy characterized by specific morphological features on cardiac MRI (CMR).^1^ Fewer than 50 cases have been reported worldwide, showing a wide spectrum of clinical presentations that make diagnosis challenging.^2^ In the absence of standardized management guidelines, therapy remains individualized. We report a case of a middle-aged woman with atypical symptoms, in whom detailed CMR evaluation led to the incidental diagnosis of ILVAH, followed by appropriate medical management and follow-up.

## Summary figure

**Figure:**
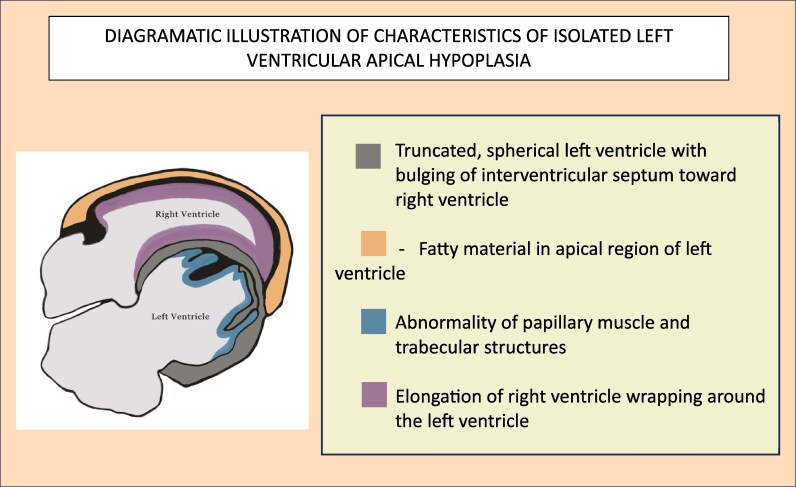


## Case presentation

**Figure 1:**
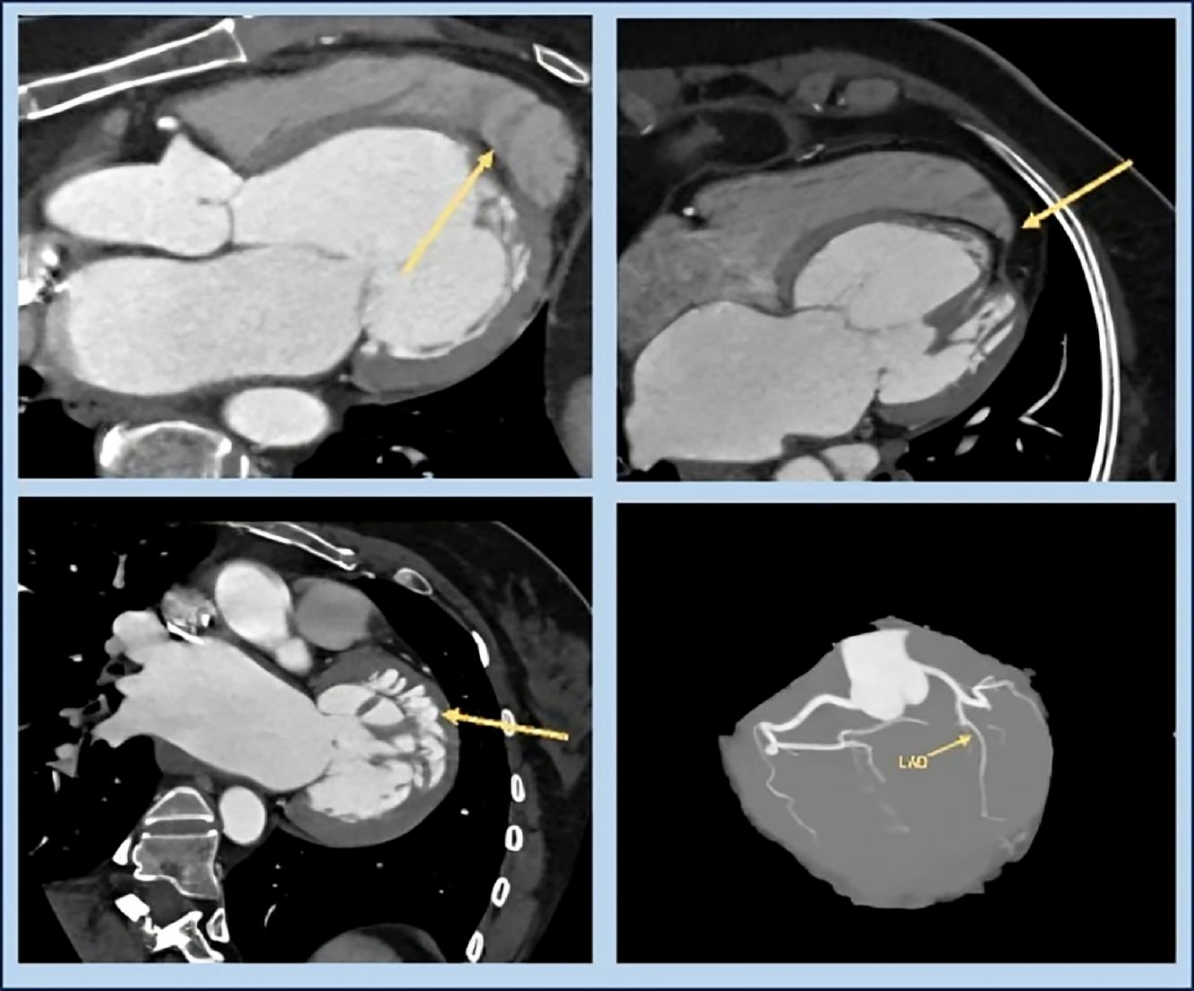
CT coronary angiogram images suggestive of (*A*) wrapping around the right ventricle over the deficient left ventricle apex, (*B*) fatty infiltrating hypoplastic left ventricle apex, (*C*) abnormal origin of papillary muscles and hypertrabeculation, and (*D*) normal coronary arteries with Type I left anterior descending artery.

**Figure 2:**
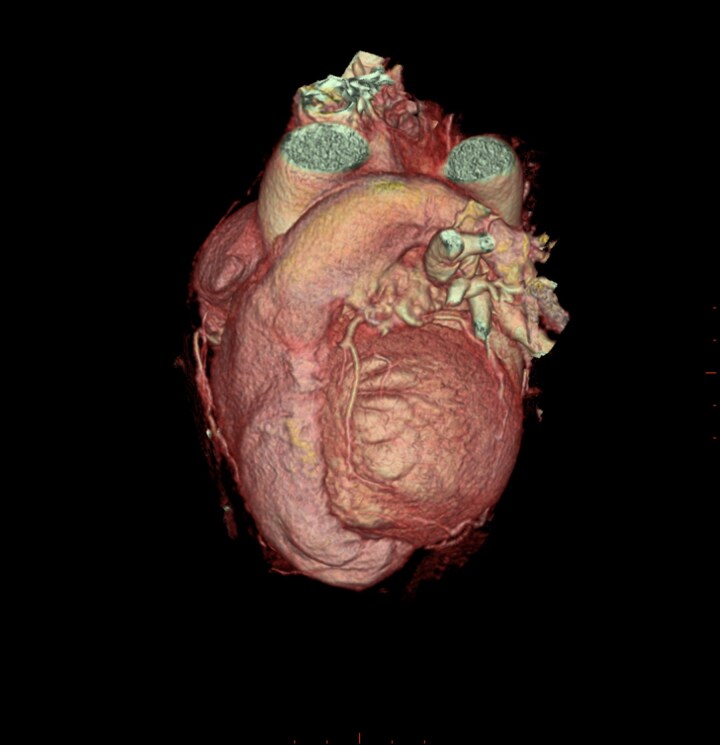
VRT—MDCT image of the heart showing truncated left ventricle apex, wrap-around right ventricle, and Type I left anterior descending artery.

A 43-year-old woman presented with mild, post-prandial chest discomfort and dyspepsia, unrelated to exertion and without dyspnoea. She had no comorbidities or family history of ischaemic heart disease or sudden cardiac death. Baseline investigations, including cardiac troponin and chest X-ray, were normal. Electocardiogram (ECG) showed normal sinus rhythm with left anterior fascicular block. Transthoracic echocardiography revealed severe hypokinesia in the left anterior descending artery (LAD) territory, dilated left atrium, mild mitral regurgitation, and a left ventricular ejection fraction (LVEF) of 35%. A CT coronary angiogram revealed a Type I LAD with right dominance, as well as dilatation of the left atrium and ventricle, and focal thinning at the apex of the left ventricle (*Figure 1* and 2). The patient was subsequently advised to undergo conventional coronary angiography.

**Figure 3:**
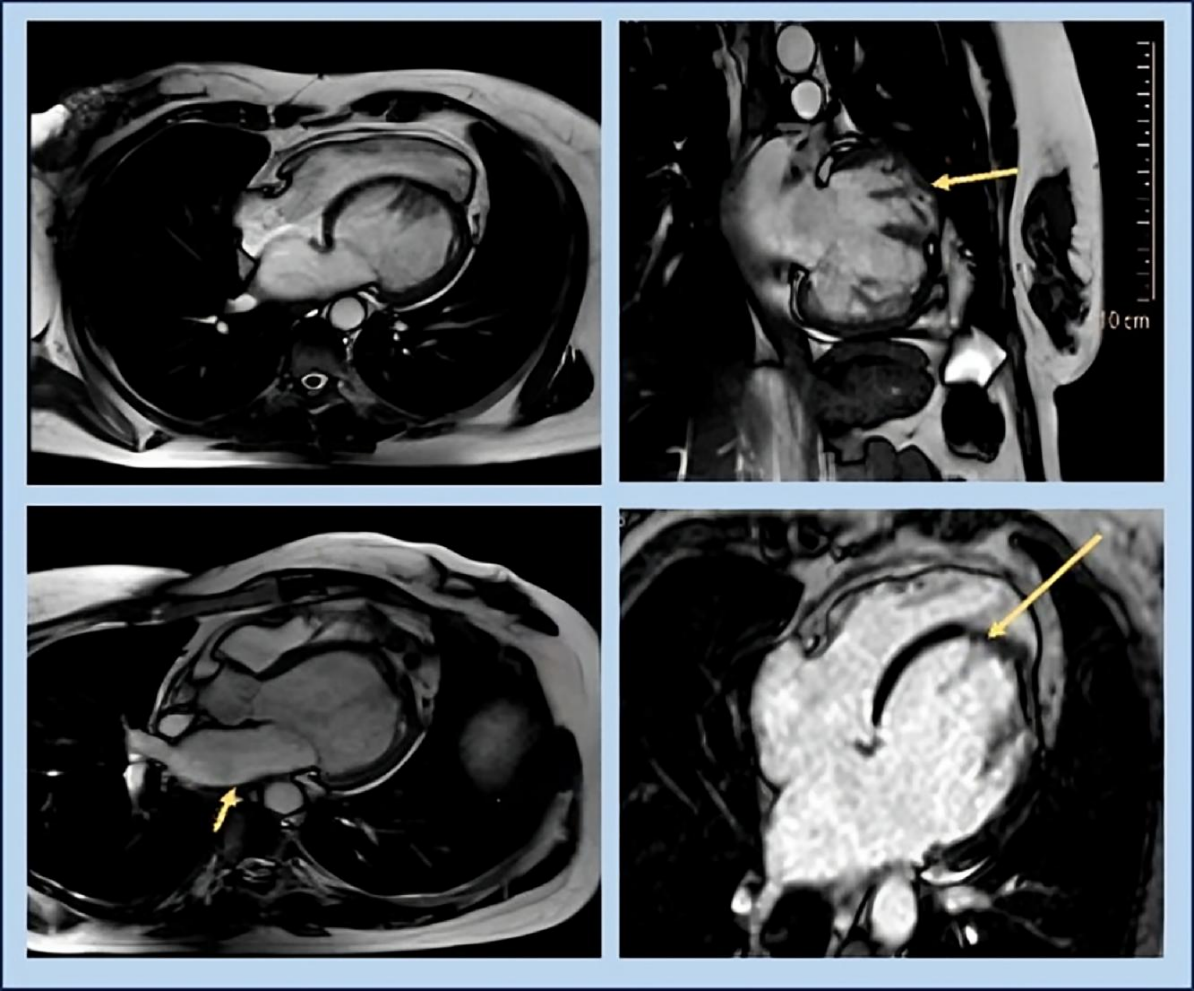
Cardiac MRI images suggestive of (*A*) cine 4 CH SSFP image that revealed globular and truncated left ventricle with elongated right ventricle which wraps around the deficient left ventricle apex and IVS bulging towards the right ventricle, (*B*) complex pattern of papillary muscles with bifid anterior and posterior papillary muscles with normal chordae attachment, (*C*) mildly dilated left atrium, and (*D*) delayed enhancement IR-GRE 4CH image showing no delayed myocardial hyperenhancement.

Seeking a second opinion, she presented to our institute a week later and was on sacubitril–valsartan, eplerenone, metoprolol, and dapagliflozin. Given the discrepancy between clinical presentation and the investigations, a CMR was performed. The CMR (*Figure 3*, Supplementary video) revealed characteristic features of isolated left ventricular apical hypoplasia, with a normal left ventricular ejection fraction of 58%, the left ventricle (LV) end-diastolic volume was 109 mL, and the end-systolic volume was 45 mL. The LV appeared mildly dilated and possessed a globular shape, with severely hypoplastic apical segments. The mitral annulus was of normal size, showing no significant regurgitant jet. The papillary muscles exhibited a complex bifid pattern of origin on the trabeculae from both the anterior and posterior walls of the left ventricle. The right ventricle (RV) appeared normal in size, elongated, and wrapped around the absent apex of the left ventricle. Notably, there was epicardial fat invagination at the site of the deficient LV apex. Crucially, no delayed myocardial enhancement was detected in either the left or right ventricles, indicating the absence of infarction, fibrosis, or infiltrative myocardial disease.

## Discussion

Isolated left ventricular apical hypoplasia is a rare and unclassified cardiomyopathy that was first recognized as a distinct clinical entity in 2004.^3^ It is characterized by specific features observed on CMR, including:

- A truncated and spherical LV with bulging of the interventricular septum towards the RV
- Accumulation of fatty material in the apical LV
- Anomalies involving the papillary muscles or the trabecular architecture
- Elongation of the RV, which encircles the deficient apex of the LV^1^

Anatomically, ventricular development can be understood through three components—inlet, apical trabecular, and outlet—rather than the traditional ‘sinus’ and ‘conus’.^4,5^ Anomalies affecting one component may result in isolated apical hypoplasia, while more diffuse involvement produces hypoplastic left heart syndrome. This conceptual framework, initially described by Goor and Lillehei and later refined by Freedom and Anderson, helps contextualize ILVAH as hypoplasia of the LV’s apical trabecular component.^5^ The condition is hypothesized to be congenital, possibly due to abnormal ventricular partitioning during embryogenesis, leading to a spherical LV and elongated RV.^1^ Preliminary studies have explored potential lamin A/C gene involvement, though no definitive genetic correlation has been established.^2,6,7^ An ongoing trial is assessing whole-exome sequencing in a small ILVAH cohort.^1^ Current evidence supports a sporadic origin, with no definite hereditary or familial predisposition. The clinical presentation of ILVAH can vary significantly, ranging from asymptomatic patients to those experiencing symptoms such as fatigue, exertional dyspnoea, palpitations, chest pain, and syncope. In a systematic review of 37 cases, breathlessness was the most common presenting symptom, noted in 40.5% of patients, with a mean age of diagnosis at 26.1 ± 19.6 years. Typical ECG findings included T-wave abnormalities and right axis deviation, frequently accompanied by poor R-wave progression. Atrial fibrillation or flutter was identified in 24.3% of the cases. Given the potential for progression, patients with ILVAH should be monitored for signs of heart failure, pulmonary hypertension, and arrhythmogenic complications. Management strategies remain poorly defined due to the scarcity of reported cases; however, current protocols typically recommend heart failure therapy as the initial treatment approach, with some patients necessitating device therapy, such as biventricular pacing.^8^

Differential diagnoses for ILVAH include hypoplastic left heart syndrome, characterized by underdevelopment of the aortic valve and artery as well as the entirety of the LV, often with stenotic or atretic mitral valves. Left ventricle non-compaction, which presents as a diffusely enlarged LV with a prominent trabeculated endocardium giving it a ‘spongy’ appearance, can arise from disrupted endomyocardial morphogenesis. Congenital LV dysplasia, with or without right ventricular dysplasia, should also be considered. Notably, these conditions tend to be identified earlier in life, whereas ILVAH is frequently diagnosed during adolescence or adulthood, often incidentally or in the context of acute heart failure.^1^

Thromboembolic risk may be attributed to the presence of atrial fibrillation/flutter, left ventricular mural thrombus, and severe left ventricular dysfunction as reported in a few cases; however, no clear evidence confers an intrinsic risk in patients with ILVAH thus far.^1,8^ Existing literature demonstrates normal coronary anatomy in the majority of ILVAH cases. However, a few reports describe apparent displacement of the LAD, likely due to the truncated left ventricular apex and altered ventricular geometry rather than a true coronary anomaly.^9^While no formal guidelines exist, management generally mirrors standard heart failure therapy, with device implantation reserved for patients with systolic dysfunction or arrhythmias.

In our patient, atypical chest pain led to the incidental diagnosis of ILVAH. Initial echocardiography underestimated LVEF, likely due to the hypoplastic apex. Although anti-heart failure therapy was initiated after her initial discharge, the short duration makes it unlikely that the observed improvement in LVEF was medication related. It is therefore plausible that the initially reduced LVEF on 2D echocardiography reflected technical underestimation due to the hypoplastic apex. Our patient was maintained on guideline-directed medical therapy (sacubitril–valsartan 100 mg twice daily, eplerenone 25 mg once a day, metoprolol 25 mg once a day, and dapagliflozin 10 mg once a day) despite a preserved LVEF on CMR, in view of the potential for progressive ventricular dysfunction and heart failure described in patients with ILVAH. Follow-up echocardiograms at 3 and 6 months demonstrated normal LVEF and no pulmonary hypertension or arrhythmias.

## Conclusion

This case highlights the importance of recognizing ILVAH as a distinct cardiomyopathy with characteristic imaging findings. Long-term follow-up focusing on ventricular function and rhythm surveillance is essential. Further studies are needed to clarify the genetic basis, natural history, and optimal management of ILVAH.

## Lead author biography


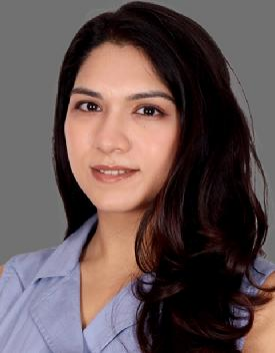


Dr Ujjwala Tulluri is a cardiology resident at Asian Heart Institute, Mumbai, India. She has completed her MD Internal Medicine from M. S. Ramaiah Medical College in Bangalore, India, in 2021.

## Supplementary Material

ytaf650_Supplementary_Data

## Data Availability

The data underlying this article are available in the article and in its online Supplementary material.
